# Design and Testing of a Co-Rotating Vibration Excitation System

**DOI:** 10.3390/s19010092

**Published:** 2018-12-28

**Authors:** Angelos Filippatos, Tino Wollmann, Minh Nguyen, Pawel Kostka, Martin Dannemann, Albert Langkamp, Loic Salles, Maik Gude

**Affiliations:** 1 Institute of Lightweight Engineering and Polymer Technology (ILK), Technische Universität Dresden, 01307 Dresden, Germany; tino.wollmann@tu-dresden.de (T.W.); minh.nguyen@tu-dresden.de (M.N.); pawel.kostka@tu-dresden.de (P.K.); martin.dannemann@tu-dresden.de (M.D.); albert.langkamp@tu-dresden.de (A.L.); maik.gude@tu-dresden.de (M.G.); 2 Department of Mechanical Engineering, Imperial College London, Exhibition Road, London SW7 2AZ, UK; l.salles@imperial.ac.uk

**Keywords:** vibration excitation, rotor, structural dynamic behaviour, rotational testing

## Abstract

A vibration excitation system (VES) in a form of an active coupling is proposed, designed and manufactured. The system is equipped with a set of piezoelectric stack actuators uniformly distributed around the rotor axis and positioned parallel to each other. The actuator arrangement allows an axial displacement of the coupling halves as well as their rotation about any transverse axis. Through the application of the VES an aimed vibration excitation is realised in a co-rotating coordinate system, which enables a non-invasive and precise modal analysis of rotating components. As an example, the VES is applied for the characterisation of the structural dynamic behaviour of a generic steel rotor at different rotational speeds. The first results are promising for both stationary and rotating conditions.

## 1. Introduction

New developments in the turbo-machinery industry lead to constantly increasing demands on the economic and ecological efficiency and on the reliability of rotating components. However, the required increased performance of those components leads to higher dynamic requirements, which can be achieved with a better understanding and characterisation of the dynamic behaviour under rotating conditions.

In particular for the development of high performance rotors such as fan blades of aircraft engines and turbine shafts for steam turbines a thorough analysis and understanding of the dynamic characteristics are of high relevance. In this context, a defined vibration excitation is an important prerequisite for the accurate, in-depth experimental determination of the structural dynamic behaviour of rotors. An analysis of the natural frequencies, modal damping and shapes is essential for the development of high performance rotors and can also be included in the monitoring of their structural state. In most cases the operational rotor excitation cannot fulfil the requirements for such an analysis. Since these properties are greatly dependent on the rotational speed, an excitation method of different degrees of freedom in a co-rotating coordinate system is of particular interest.

### 1.1. Motivation

Unbalanced, misaligned and mistuned components can cause vibrations which could lead to undesirable dynamic load on the system, flow separation and turbulence. This is especially evident in the case of aero-engine turbines and power energy systems.

In addition, the operation at critical speeds is always associated with increased vibration amplitudes that can lead to unsteady flow conditions and an accompanying increased sound radiation. Therefore, a characterisation of the dynamic behaviour of rotors under different rotational speeds is an important factor for the optimal design and safe operation of rotors. Specifically, new experimental methods appropriate for the investigation of complex phenomena such as non-linear behaviour, gradual damage behaviour, internal friction and damping describing the structural behaviour of metallic and composite rotors are required [1,2]. These phenomena are not adequately considered in existing simulation models as they cannot be fully captured with existing measurement techniques not least due to a lack of suitable vibration excitation systems [3].

### 1.2. Aim and Outline of the Paper

The aim of the described investigation is to develop a novel excitation method enabling an on line vibration excitation for a non-invasive experimental modal analysis. To achieve this, a vibration excitation system is proposed that can be utilised for an online aimed excitation of rotors. Based on external sensor system and control software, a set of co-rotating piezo-stack actuators integrated to the VES is excited in a predefined way. The novelty here is that the piezo-stack actuators are positioned parallel to the rotational axis and they rotate along with the investigated rotor, which is an experimental approach that has not yet been reported.

The paper is divided as follows. In Section 2 the concept is presented and the requirements are identified for a specification of the concept. Based on the results, the dimensioning of the most important components of the system is shown.

In Section 3, the prototype development and the assembly of the piezo-stack actuators are described. Based on the developed prototype, first tests under rotation are conducted in Section 4 in order to assess the functionality of the system and to perform an experimental modal analysis for a metallic generic rotor.

### 1.3. State-of-the-Art

For the experimental modal analysis of rotating components several methods are known. These methods combine different excitation and measurement techniques for the identification of modal properties either at a standstill or at rotating conditions.

#### 1.3.1. Excitation Systems

The excitation methods for rotors can be classified according to the position of the excitation, resulting either in stationary excitation or in a co-rotating excitation. Several excitation methods are state-of-the-art. These include air jets, electromechanical shakers, active magnetic bearings, piezo-stack actuators, permanent magnets, non-contact laser ultrasonic excitation, eddy current systems and macro fibre components (MFC) [4,5,6,7,8]. Other applications include the use of piezoelectric materials both as actuator and as sensor, specifically for some controlling applications [9,10]. For example permanent magnets have been used to excite titanium blades at fixed engine orders [11,12] and macro fibre composites were used at scaled metal and composite fan blades providing in both cases relative small forces [13,14,15]. Electromagnetic excitation can also be used for the excitation or damping of the shaft or of a test rig itself, as presented in Refs. [16,17]. An application at a rotary device having piezoelectric stack actuators to reduce vibrations of the rotating component, could also be used for the excitation of a rotating component [18].

In general, for a non-invasive base excitation the main disadvantage is the low force provided, e.g., by active magnetic bearings, in comparison to the force provided by piezo-stack actuators [19]. The implementation of a co-rotating excitation method into a system involves great challenges due to the rotational forces. The integration of an actuator, e.g., piezo elements to the rotor results also in changes of its mass and stiffness characteristics, as well as it requires a complex integration and contacting of the actuators [20]. A replacement of the actuators after a failure is also not possible. Even solutions implementing piezo stack actuators which are perpendicular to the rotor bearings lack the necessary degrees of freedom for the excitation in axial direction [21].

#### 1.3.2. Methods for Vibration Measurement on Rotating Components

For the vibration measurement of rotating structures several systems are available. A well-known non-contact measurement method based on the stationary coordinate system is the blade tip-timing method, which evaluates time-dependent differences of blade passages after multiple rotor revolutions, a known method first used in 1970’s, [22,23,24,25], as well as using continuous-scanning laser doppler vibrometry (LDV) [26].

Another standard measurement approach is the application of strain gauges and lately fibre Bragg grating at rotors, which measure the vibration characteristics continuously during the rotation. The strain gauges are attached to points of reference at selected blades where frequencies and amplitudes of blade vibrations are determined by measuring strain dynamic components. However, the static position of the blades, which can provide significant diagnostic information about the possible damage of the blade, cannot be determined with strain gauge systems. Another drawback is the fact that in the aggressive environment of the steam turbine, the strain gauges experience a short lifetime of usually several days or weeks. In addition, such systems can monitor only selected blades [23].

The vibration measurement of a rotating component with a coupled system of a scanning laser vibrometer and an optical derotator delivers confident results [27], enabling a non-contact determination of the modal properties. The laser beam is directed using a rotating, coaxially mounted Dove prism to the rotor and thus it can measure in a co-rotating coordinate system [28]. However, limitations are evident as an alignment of the derotator in axial direction to the plane of rotation is necessary. Moreover the optical derotator requires a full optical window to the rotating component, making it more suitable for laboratory conditions and prototype testing.

The optical derotator constitutes an autonomous device whose main elements are a rotating unit with a Dove prism, a precision driving and an adjustment unit [29]. On the adjustment unit the scanning laser doppler vibrometer as well as a single-point laser doppler vibrometer are mounted, where defined scan points are measured sequentially. The single-point laser doppler vibrometer serves as an accurate phase reference for the reconstruction of the vibration modes. Both lasers are directed towards the rotating object through the Dove prism and they follow the defined measurement point of the target precisely synchronised by means of the rotation optics [30].

## 2. Concept and Design of the Excitation System

**Figure 1:**
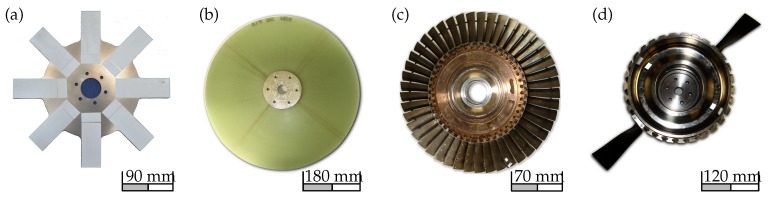
Four possible rotor geometries that can be measured using the developed system; (**a**) a generic steel rotor, (**b**) a composite rotor, (**c**) an industrial steel rotor and (**d**) a titanium disk with composite blades [15,32,33,34].

A versatile excitation system, suitable for investigations on different rotors is designed in a multi-material development approach dealing with the entire material, design, simulation, assembly and prototype testing chain. Requirements are identified from a previous concept [31] for selected rotor components as input for the design phase as shown in Figure 1. The concept specification takes place for these rotors and their project-related requirements. A detailed design concept of a prototype is proposed that is subsequently manufactured and tested.

### 2.1. Principal Concept

The demand of an online vibration excitation for the experimental investigations of different metallic and composite rotors has led to the concept of a co-rotating active coupling system and to a patent application [31]. The patent describes a device that can excite rotors, such as centrifuges, aircraft engines or turbines to vibrate, compensate imbalances and actively damp selected vibration modes during operation, i.e., under rotation.

**Figure 2:**
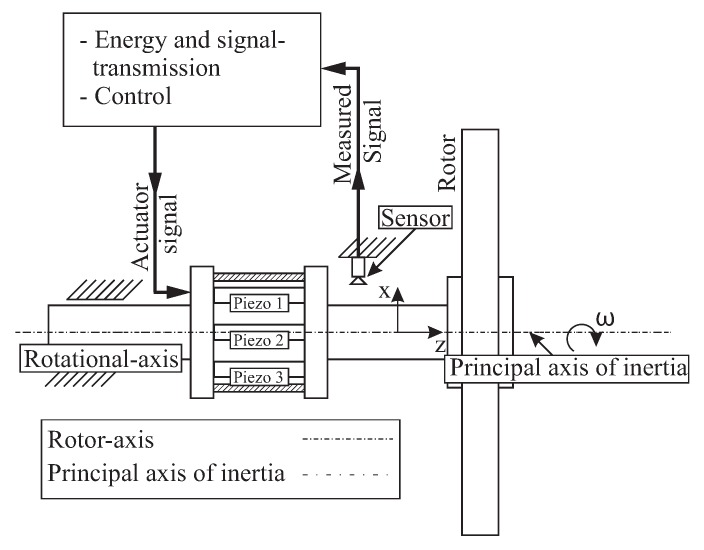
Principal concept of the excitation system with sensors, actuators and software [31].

Here this concept is further elaborated and a co-rotating vibration excitation system is designed and equipped with a number of piezoelectric stack actuators uniformly distributed around the rotor axis and positioned parallel to each other, connecting two coupling halves Figure 2. The actuator arrangement allows an axial displacement of the coupling halves as well as their rotation about any transverse axis. The applied piezoelectric actuator system is particularly suitable for the intended test rotors Figure 1a due to the appropriate degrees of freedom and the possibility of high force generation in a broad frequency range. This is accomplished using three piezo-stack actuators, which are evenly distributed over a common perimeter. The power supply to the actuators is realised through a suitable high voltage slip ring.

### 2.2. Requirement Identification and Selection of Technical Parameters

The requirements for the development of the excitation system depend on the rotors’ boundary conditions, on the used test bench system and on the requirements from the existing scientific projects. Since the system is based on an existing design example, some requirements are to be considered as base features that only vary in their values. For example, the necessary preload of the actuators can be achieved through various design solutions. In addition, the coupling elements between the VES, rotor and test rig can be modified for each type of rotor - test rig combination, respectively.

The most important **requirements** for the design of the VES are listed below. First of all, the **geometry** must be as compact as possible, with a short length to reduce unbalance and bending of the VES. Furthermore, the VES kinematics have to be very accurately defined in the axial direction (z) and to tilting and pivoting (rotation x, y) due to high acceleration values. The occurring **forces** have to be analysed, originating from the preload force of the piezo actuators and forces from existing imbalance. Furthermore, a proper selection of actuators that enable sufficiently high actuation forces for a reliable measurement of the rotor’s vibration response is required. A suitable **material selection** for the VES is also important for achieving an overall low mass combined with sufficient stiffness. This goal could be met by the application of composite material with high specific anisotropic stiffness and strength.

Beyond the above-mentioned basic requirements, additional requirements exist to assure for the proper operation of the VES. For example, a **strain output** is required from each actuator for the determination of the prestress and actuator position and has to be taken into consideration during the design phase. Furthermore, a **redundant safety solution** has to be designed and implemented for high static and dynamic loading within actuation range. Last but not least, **manufacturability** with small tolerances to reduce imbalance has to be taken into consideration during the design phase as well as a simple **assembly** with central cable feed.

Based on the given requirements the most important technical parameters are identified and taken into consideration during the design phase in order to ensure a proper functionality of the system. The maximum **rotational speed** is an important parameter since it determines the acting loads caused directly or indirectly by centrifugal forces. Another parameter is the maximal transmitted **drive torque**, resulting from the required angular acceleration and the moment of inertia of the coupled rotors. The **rotor mass** distribution determines the magnitude of the moment of inertia and the required bending stiffness of the entire system. Under dynamic load the **preload force** is necessary to protect the actuators from damage that can be caused by the inertia of their own and coupled masses. Another important parameter is the **axial rigidity** (along the stroke path) of the preload system, which determines the residual stroke and characteristic curve of the piezo actuator and should be a maximum of 30% of the actuator stiffness [35]. The value results overall from the selected material and the specific design. In terms of **assembly space**, the diameter of the cable bushing is important, as the cables must be routed centrally along the axis of rotation in order to minimise possible sources of imbalance.

### 2.3. Concept Specification

**Figure 3:**
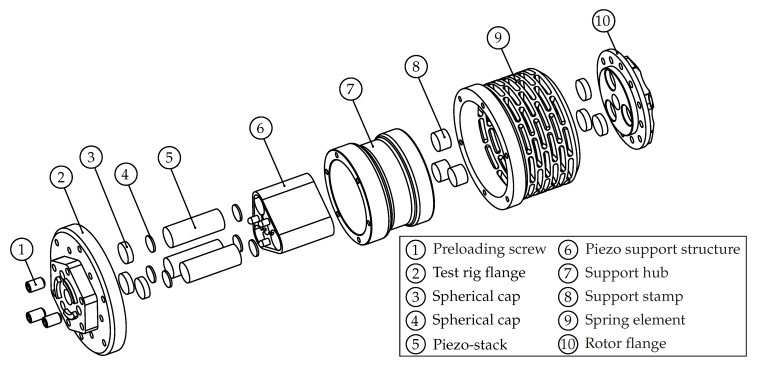
An exploded design view of the main components of the vibration excitation system.

The formulated requirements are solved by the proposed vibration excitation system, which is equipped with a set of piezoelectric stack actuators, Figure 3. The main components of the excitation system are the **piezo actuators**, the metallic elastic **coupling element** and the **carbon fibre-reinforced epoxy bandaging** of the actuators. The main configuration is a parallel connection of the actuators with one another forming an actuator group, and with a parallel connection of the actuator group with the elastic coupling element.

The actuators are uniformly distributed around the rotor axis of rotation and are positioned parallel to each other. This arrangement allows both a high overall torsional stiffness (main power flow) and a favourable actuator pre-stress, which is characterised by a low stiffness of the coupling’s pre-load system. The arrangement also allows an axial displacement of the coupling elements as well as their rotation about any transverse axis. To allow a multi-directional excitation, the metallic elastic coupling element (8) is designed as a cylindrical steel hub incoorporating multiple flexure hinges [36], which provides direction-dependent adjustment of the stiffness. The piezo actuators (5) that are used for the excitation exhibit a very limited bending strength. During operation the actuators are exposed to high centrifugal loads, that act perpendicular to the central axis, causing bending loads. In order to ensure proper operation, the actuators are embedded in a support structure (6), which consists of an aluminium core that was wrapped up by a carbon fibre-reinforced epoxy bandage and positioned between spherical elements (2, 3). Bending loads throughout the balancing elements are prevented by the support hub (7), which includes 3 symmetrically distributed holes, that act as slide bearings.

For the operation of the piezo actuators, an excitation signal has to be generated in an external programmable signal generator and then enhanced by an amplifier. The acquisition of the necessary signals is performed by an external sensing system. In the described investigations, the Laser scanning Doppler vibrometer PSV-I-400 from the company Polytec [30] is used.

#### 2.3.1. Selection of Piezo-Stack Actuators

**Figure 4:**
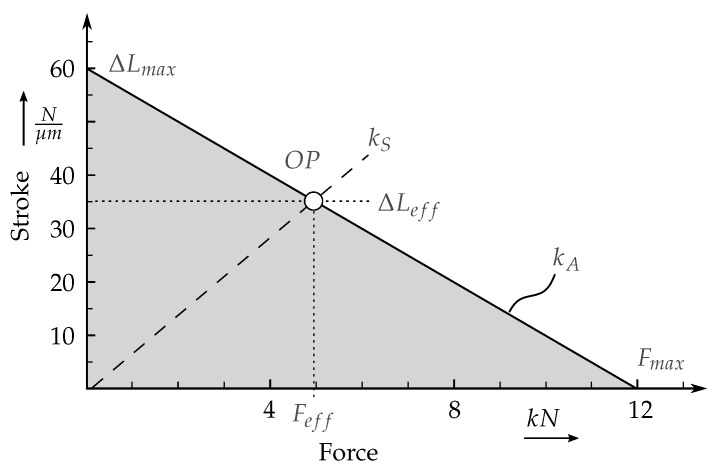
Force/stroke diagram of the piezo actuator PST1000/16/60, its stiffness and intersecting stiffness defined by an exemplary mechanic (S), according to Ref. [35].

Piezo stack actuators can be applied to act as linear electromechanical drivers [35]. They are mostly used as an expanding element, which is often called a pusher, which generates a compressive force. The applied piezo stack actuators are composite structures that are made by connecting multiple piezoceramic discs with typical maximal voltages ranging from 300 V to 1000 V. The discs are made from electro-active piezoceramic material, most commonly named PZT, a material combination of lead, zirconium and titanium mixed-oxides. The complete motion cycle is almost proportional to the input of the voltage signal from the direct current up to high frequencies. Based on the dynamic requirements for the vibration and the identification of the rotor eigenfrequencies, specific actuators could be selected, that can operate in the required bandwidth. The amplitude of excitation can be controlled from the given voltage signal input. As long as the investigated structures and the applied actuators ar in the linear area, then there should be a linear dependency between amplitude of excitation and amplitude of the eigenfrequencies. Non-linear systems behave differently as they are dependent of the excitation amplitude and consequently from the resulting vibration amplitude. In this manuscript only linear systems have been in the scope of investigation. The former mentioned pre-loading of the actuators is necessary, if high frequencies or highly changing loads have to be achieved. In this case, the inertia of the actuator itself can cause cracking or delamination between several discs. Therefore, the stiffness characteristics of an actuator have to be compared to the stiffness of the pre-load via the force/stroke diagram, such as the one shown in Figure 4. For this purpose, the required maximum force $F_{max}$ and the maximum stroke $\Delta L_{max}$ on the corresponding axes are transferred into a force-stroke diagram and these points are connected via a straight line. This results in the stiffness characteristic of the actuator. The area below the straight line represents the theoretical working range of an unloaded actuator. For the determination of the suitable operating point, an origin line $k_{s}$ is entered whose slope corresponds to the stiffness of a spring load. The point of intersection of the straight line of load stiffness with the actuator stiffness characteristic marks the operating point. The frequency range of application depends on the first eigenfrequency of the actuator and the excitation range of the used amplifier. In the typical range of application for each actuator, which is normally given form the manufacturer, the force is mainly dependent from the applied voltage signal and the energy supplied.

Hereby, OP marks the operating point corresponding to the maximal actuator voltage, $\Delta L_{eff}$ represents the maximum of stroke and $F_{eff}$ its corresponding force. It has to be noted, that the corresponding force represents the lose in maximum achievable force $F_{res}$, which is also minimized by static forces $F_{stat}$ and leads to the following equation

(1) $$F_{res}=F_{max}-F_{eff}-F_{stat}$$

An actuator is suitable if its stiffness ($k_{A}$) is above or coincides with the load. Based on the requirements, a PST 1000/16/60 actuator from Piezosystem Jena GmbH was selected as it provided a good balance between the force-displacement profile and the possible assembly space. To introduce the pre-load forces to each actuator, 3 screws are positioned right behind the load balancing elements and an appropriate tightening torque is applied to these screws.

#### 2.3.2. Dimensioning of the Coupling Element

**Figure 5:**
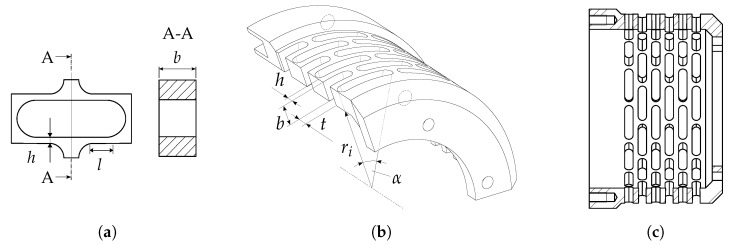
(**a**) Sketch of a single spring element (flexure hinge); (**b**) Geometric parameters; (**c**) CAD drawing of the coupling element.

For the design of the coupling element a parametrised computer-aided design (CAD) model is used in order to adapt the stiffness. The decisive parameters for the design process are the width *b*, height *h* and length *t* of the spring component, as shown in Figure 5b. The design goal is to achieve an axial stiffness of the prestress-system which is equal to 10% of the actuator’s stiffness, resulting in a low reduction of its stroke. At the same time the stress fields of the spring element were to remain in acceptable limits using a safety factor of 1.5. Then the coupling is modelled to be clamped on one side and loaded with a force on the opposite side, which is described in details in Section 2.4. The ratio of force to the resulting displacement difference gives the stiffness for different geometry configurations.

### 2.4. Simulation-Assisted Prototype Design

A numerical simulation of the excitation system was performed by means of a finite element model. The system was modelled in the commercial software ANSYS Workbench with the help of ANSYS Composite PrepPost. Selected degrees of freedom were constrained in order to simulate fixed boundary conditions at the rig adapter at position (2) Figure 3. Furthermore, a number of simplifications were implemented during the design process of the simulation model. The bolt connections were not modelled since the focus was on the core section including the piezo stack actuators, the composite bandage and the elastic spring element. The piezo actuators were further modelled as solid piezo ceramic elements, without taking their layered structure into account. The stress distribution at the excitation system is investigated under a preload of 4000 N on each of the piezo elements and at a rotational speed of 15,000 rpm. The main focus was on the carbon fibre-reinforced plastic (CFRP) bandage and its influence on the deformation behaviour of the piezo elements. Another focus was on the elastic coupling element, since this part was designed to be the most flexible part and thus experiences the largest strains and stresses.

#### 2.4.1. Dimensioning of the CFRP-Bandaging

**Figure 6:**
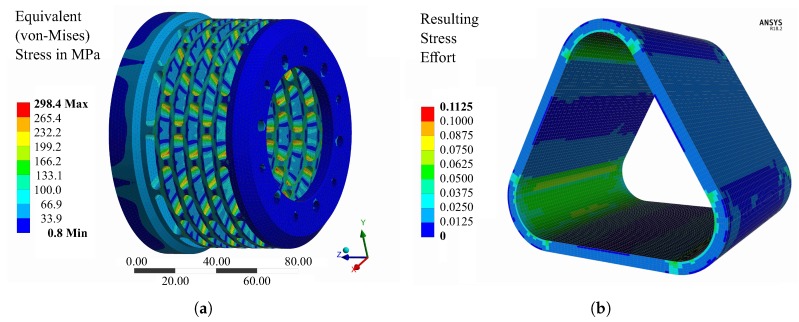
Stress distribution of the metallic spring element (**a**) and the resulting effort of the CFRP bandage (**b**) under centrifugal load at 15,000 rpm and pre-load of 4000 N at each piezo element.

The stress distribution for both the piezo actuators and the CFRP-bandage are numerically investigated using the same boundary conditions as previously presented. The piezo elements are wrapped with the CFRP bandage to reduce the bending caused from the centrifugal forces. The filament wound bandage was modelled with extruded Solid186 elements where six layers are used to represent the composite material. For the evaluation, the stress in fibre direction and transverse to the fibre direction as well as the Failure Mode Concept was used [37]. The resulting stress effort containing the combination of two fibre failure modes (tension and compression) and three matrix failure modes (tension, compression and shear) was considered and showed no failure with an uncritical value of approximately 0.1, as demonstrated in Figure 6b. An intermediate modulus carbon fiber is selected and embedded in epoxy resin providing high degree of radial stiffness. Based on the numerical results and under consideration of the technical requirements of a minimum deformation and assembly space, the composite layup consists exclusively of 0${}^{\circ }$ layers with a thickness of 3 mm.

#### 2.4.2. Elastic Coupling Element

For a load case of 4000 N, an equivalent von-Mises stress of approximately 300 MPa is located at the inside edge of the coupling element, as shown in Figure 6a. The high stresses and the required robustness of the coupling by a minimum mass dictates the use of a high-alloyed steel for the main parts of the coupling. Therefore, alloy steel 42CrMo4 was selected in order to achieve a safety factor towards operational stability of the steel material. Consequently, the mechanical integrity is also demonstrated by the simulation results for the given load case.

## 3. Manufacturing and Prototype Assembly

The actuators used here are stacks of several layers of thin piezoelectric discs, which are glued together. Due to this fact they exhibit a fragile and brittle behaviour when exposed to tensile loads parallel to the central axis and bending forces perpendicular to its central axis. Highly dynamic loads can also result in degradation of the adhesion inside the bonds. Therefore, to prevent any damage to the actuators, a preload has to be applied and the actuators are not allowed to experience any bending. After the assembly, a pre-load was applied on the piezo actuators using a screw mechanism and subsequently the actuators were checked for their functionality.

### Embedding the Piezo Actuators

**Figure 7:**
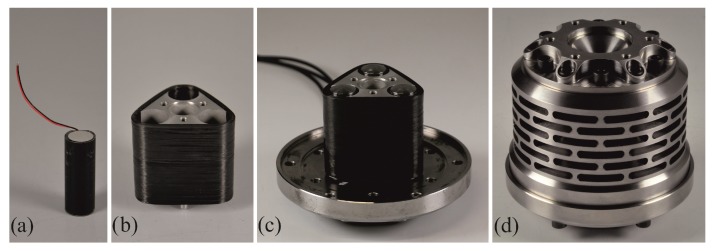
Embedded piezo actuator (**a**), filament wound piezo carrier (**b**), finished piezo mounting with integrated piezo actuators (**c**) and outside view on the VES (**d**).

The piezo actuators (PSt 1000/16/60 from Piezosystem Jena GmbH) had to be prepared for the integration in the piezo mounting system. As the piezo stack actuator has no defined outer diameter in the delivered condition, an embedding tool was used to surround the piezo element with cast resin to create a defined cylindrical surface, as shown in Figure 7a. After the curing of the resin, the piezo was removed from the embedding tool and pressed into the piezo mounting, where also the spherical steel heads are connected to the piezo actuators.

The piezo carrier as well as the piezo-stack actuators are wrapped with CFRP, shown in Figure 7c, which were wound in a wet filament winding process and cured at room temperature and tempered at 60 ${}^{\circ }$C. After curing of the resin and subsequent tempering, the component was demoulded and trimmed at the edges to remove unnecessary resin. In Figure 7d, the finished core component of the excitation system is shown, including the filament wound CFRP bandage and the spherical heads for the load transmission.

## 4. Prototype Testing

As the VES is exposed to dynamic loads, it was experimentally tested during operation in order to assess the piezo actuators. Then, the structural functionality of the whole system is investigated, in particular to examine the durability of the composite assembly and of the coupling element.

### 4.1. Endurance Test by Rotational Run-Up

**Figure 8:**
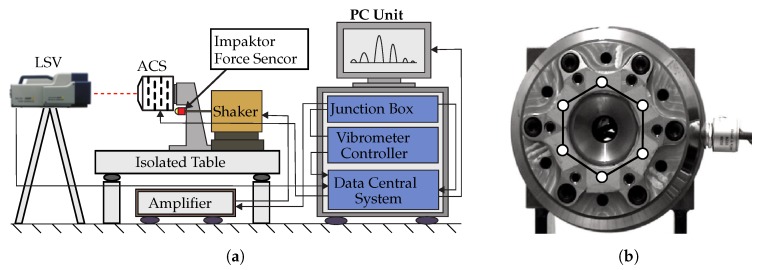
Test set-up of the modal analysis of the VES (**a**) and applied measuring grid (**b**).

At first, the system had to undergo a rotational endurance test in order to evaluate its resilience under high centrifugal loads. These loads lead to different loading conditions depending on the position and geometry of each specific part. For instance, the coupling element exhibits an expansion similar to a pressure vessel, due to the reduced stiffness in its middle section, whereas the piezo actuators exhibit bending loads, due to their eccentric position. For this purpose, the system was mounted onto a test bench and stepwise exposed to several rotational speeds. The VES has been accelerated with 2 rad/s${}^{2}$ to three different rotational speeds (5000 rpm, 10,000 rpm and 15,000 rpm), then kept on constant speed for 120 s and decelerated to a standstill. At each rotational test a visual inspection was performed in order to detect possible defects and damage initiation. Furthermore, the functionality of the integrated piezo-actuators was examined. The measured electrical capacity of the actuators remained unchanged after each test. It could be verified that both the metal parts of the VES as well as the piezo-actuators have withstood the introduced loads and the endurance of the VES was ensured. Before and after the endurance tests additional modal analyses were performed to identify changes of the modal parameters of the VES, caused by possible structural alterations. The analysis were carried out under laboratory conditions, the set-up is presented in Figure 8a. Therefore, the VES was mounted onto a vibration isolated table and excited using an impactor with integrated force sensor, that delivered the reference of the excitation signal. Figure 8b shows the measuring grid that was applied for the laser scanning process.

The results of the modal analysis are presented in Table 1 and show marginal changes of the first 5 eigenfrequencies after the endurance test.

The relative change $\tilde{\Delta }$ of the frequencies is in the range of 0 to 3.3%. The standard deviation of the measurement system for repeated testing was examined in other tests and is around 3%. Therefore, the change of the frequencies caused by the endurance test is sufficiently low to be considered as statistically not relevant.

### 4.2. Testing of a Generic Rotor

**Figure 9:**
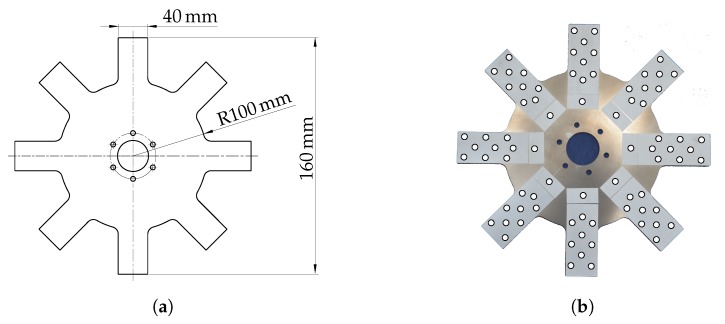
Investigated generic rotor with the main geometry parameters (**a**) and mesh grid (**b**) that was used for the modal analysis of the fan blade.

An experimental modal analysis has been performed in order to investigate the structural dynamic behaviour of the generic rotors. For this purpose, this generic steel rotor was excited and the vibration response was measured under stationary conditions and at different rotational speeds. The tests were performed under stationary conditions and during rotation up to 2000 rpm. The rotor is shown in Figure 9a together with the selected scanning grid that was used to identify the modal parameters of one single blade and the whole rotor Figure 9b.

**Figure 10:**
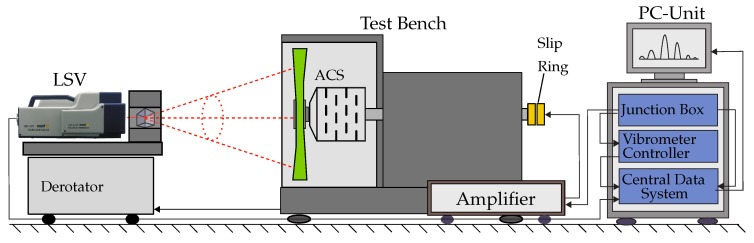
Experimental set-up for the modal analysis of rotating structures.

The acquisition of the vibration response was carried out by employing the POLYTEC laser scanning vibrometer (LSV) in combination with the POLYTEC optical derotator, that is shown in Figure 10. The derotator contains a Dove prism, driven by a motor allowing quasi-static measurements during rotation and is connected to an encoder that delivers a signal reporting the current speed of the driving spindle motor of the test bench. To generate the excitation, a high-voltage signal was transferred by a slip ring.

**Figure 11:**
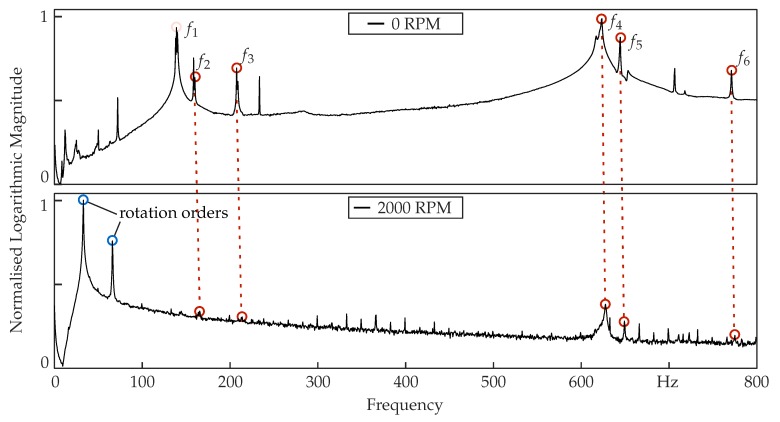
Power spectral density of the 0${}^{\circ }$ generic steel rotor at 0 rpm and 2000 rpm.

The general set-up involves the PSV-I-400 optical scanning head equipped with the highly sensitivity vibrometer sensor (OFV-505), OFV-5000 controller, PSV-E-400 junction box and the acquisition computer system. In addition, a single-point vibrometer was used to deliver a reference signal of the dynamic structural behaviour. The excitation signal was created in the PSV software and then generated and transformed by an amplifier that was driving the piezo actuators. The tests were performed under environmental conditions and under technical vacuum. During the tests, 80 measurement points were selected as shown in Figure 9b. Frequencies up to 800 Hz were measured with a resolution of 0.5 Hz. The measurements were performed at 0, 1000, 1500 and 2000 rpm. Several eigenfrequencies as well as the corresponding mode shapes were identified. The power spectral density diagrams of the generic steel rotor at two different rotational speeds are shown in Figure 11.

The analysed eigenfrequencies of the generic rotor at 0, 1000, 1500 and 2000 rpm are shown in Table 2. Increasing values of the eigenfrequencies are observed due to the stiffening effects from the rotation. Due to manufacturing tolerances, the system has undergone some inherent imbalance, which deteriorated the modal analysis and subsequently the eigenfrequency extraction in some cases. Overall, the system accomplishes its main goal, which was the excitation of a rotor under different rotational speeds along with a wide range of frequencies.

## 5. Summary and Outlook

A novel co-rotating vibration excitation system has been designed and manufactured, which allows the realisation of a non-invasive modal analysis of rotating components. The co-rotating vibration excitation system is capable of exciting rotors under rotational conditions for a determination of speed-dependent modal parameters. The results show the capability of the system under different rotational speeds on the example of a generic metallic rotor. The system was validated under non-rotating and rotating conditions using a Scanning Laser Doppler Vibrometer mounted on an optical derotator and aligned coaxially to the rotor by using a Dove prism. The novelty of this method lies to the fact that a set of piezo-stack actuators are positioned parallel to the rotational axis and they rotate along with the investigated rotor, which has not yet been reported.

A number of further investigations are under consideration and will be published in the near future. First of all, the behaviour of the excitation system should be further investigated including different types of metallic rotors and by testing real rotor-bladed discs in order to identify the application limits of the current solution. Specifically, in the case of composite rotors it is important to investigate whether the transferred energy to the rotor is sufficient due to the increased material damping and coulomb damping, e.g., between compressor blades and metal rotor hub. A further development of the high-power electronics is required in order to realise other types excitation such as sweep, sine, burst random and white noise excitation in addition to the impulse excitation used so far. Due to project and resources limitations, the current electronic system does not allow for an individual control of the piezo actuators. The required power supply of the excitation system is a challenge, as only few slip rings exist that can achieve rotational speeds up to 10,000 rpm. However they are relative expensive and also limited in the power supply. It is however possible to supply each piezo actuator using a separate channel form the same slip ring. Electrical disturbance and limitations do exist but it is possible at least under laboratory conditions. The application of the method to larger rotors supported in two or more bearings still is under investigation, with an idea of adjusting the system also for these cases.

Moreover, the operational stability as well as the fatigue of the system was not investigated, especially the behaviour of the piezo actuators and the CFRP-bandage under long-term testing and has to be also conducted. The vibration excitation system has to be compared to different excitation methods in order to determine the advantages and drawbacks of the current system. The possibility to scale up the co-rotating excitation system has to be considered for larger rotor systems and also possible limitations of hardware solutions due to complex loading conditions and high rotational speeds have to be taken into account. Last but not least, the current proposed system can be used as basis for the development of a co-rotating vibration excitation system for metallic and composite drive shafts.

In general, the proposed solution can fulfil a vibration excitation of a rotor during operation and combined with a laser scanning vibrometer mounted on an optical derotator can perform an experimental modal analysis under different rotational speeds.

## Figures and Tables

**Table 1:** Comparison of the first 5 eigenfrequencies of the VES before and after endurance testing.

	Unit	$f_{1}$	$f_{2}$	$f_{3}$	$f_{4}$	$f_{5}$
before run-up	Hz	1316	1768	2097	2987	4481
after run-up (15,000 rpm)	Hz	1316	1744	2027	2992	4476
$\tilde{\Delta }$	%	0.0	−1.4	−3.3	0.2	−0.1

**Table 2:** Comparison of the first 6 eigenfrequencies of the metallic rotor at different rotational speeds, where—are missing values due to high noise and imbalance.

Rotational Speed	Unit	$f_{1}$	$f_{2}$	$f_{3}$	$f_{4}$	$f_{5}$	$f_{6}$
0 rpm	Hz	141.0	162.0	208.0	625.0	647.0	772.0
1000 rpm	Hz	142.5	-	-	626.0	646.5	773.5
1500 rpm	Hz	143.5	-	210.5	626.5	647.5	773.5
2000 rpm	Hz	-	166.0	213.0	629.0	650.0	776.0

## Abbreviations

The following abbreviations are used in this manuscript:

VES	Vibration Excitation System
MFC	Macro Fibre Component
LDV	Laser Doppler Vibrometry
PZT	Pb(lead)–Zr(zirconium)–Ti(Titanium) mixed-oxides
CAD	computer-aided design
CFRP	carbon fibre-reinforced plastic
LSV	Laser Scanning Vibrometer

## References

[1] Gude M., Filippatos A., Langkamp A., Hufenbach W., Kuschmierz R., Fischer A., Czarske J. (2015). Model assessment of a composite mock-up bladed rotor based on its vibration response and radial expansion. Compos. Struct..

[2] Filippatos A., Gude M. (2018). Influence of Gradual Damage on the Structural Dynamic Behaviour of Composite Rotors: Experimental Investigations. Materials.

[3] Filippatos A., Langkamp A., Gude M. (2018). Influence of Gradual Damage on the Structural Dynamic Behaviour of Composite Rotors: Simulation Assessment. Materials.

[4] Pesaresi L., Salles L., Elliott R., Jones A., Green J., Schwingshackl C. (2016). Numerical and experimental investigation of an underplatform damper test rig. Appl. Mech. Mater..

[5] Raubenheimer G., van der Spuy S., von Backström T. (2013). The Use of Air Injection Nozzles for the Forced Excitation of Axial Compressor Blades. Int. J. Turbo Jet Eng..

[6] Niezrecki C., Brei D., Balakrishnan S., Moskalik A. (2001). Piezoelectric actuation: State of the art. Shock Vib. Dig..

[7] An Y.K., Park B., Sohn H. (2013). Complete noncontact laser ultrasonic imaging for automated crack visualization in a plate. Smart Mater. Struct..

[8] Ruffini V., Nauman T., Schwingshackl C. (2017). Impulse excitation of piezoelectric patch actuators for modal analysis. Topics in Modal Analysis & Testing, Volume 10.

[9] Giorgio I., Culla A., Del Vescovo D. (2009). Multimode vibration control using several piezoelectric transducers shunted with a multiterminal network. Arch. Appl. Mech..

[10] Lumentut M., Howard I. (2015). Effect of shunted piezoelectric control for tuning piezoelectric power harvesting system responses—Analytical techniques. Smart Mater. Struct..

[11] Provenza A.J., Duffy K.P. Experimental Methodology for Determining Turbomachinery Blade Damping Using Magnetic Bearing Excitation and Non-Contacting Optical Measurements. https://ntrs.nasa.gov/archive/nasa/casi.ntrs.nasa.gov/20100039314.pdf.

[12] Pešek L., Vaněk F., Bula V., Cibulka J. (2011). Excitation of blade vibration under rotation by synchronous electromagnet. Eng. Mech..

[13] Duffy K.P., Choi B.B., Provenza A.J., Min J.B., Kray N. (2013). Active piezoelectric vibration control of subscale composite fan blades. J. Eng. Gas Turbines Power.

[14] Kostka P., Holeczek K., Filippatos A., Hufenbach W. Integration of health monitoring system for composite rotors. Proceedings of the 18th International Conference on Composite Materials (ICCM18).

[15] Ruffini V., Schwingshackl C., Green J. Experimental and analytical study of Coriolis effects in bladed disk. Proceedings of the ASME 2015 International Design Engineering Technical Conferences and Computers and Information in Engineering Conference.

[16] Brown G.V., Kielb R.E., Meyn E.H., Morris R.E., Posta S.J. (1984). Lewis Research Center Spin Rig and Its Use in Vibration Analysis of Rotating Systems.

[17] Das A., Nighil M., Dutt J., Irretier H. (2008). Vibration control and stability analysis of rotor-shaft system with electromagnetic exciters. Mech. Mach. Theory.

[18] Scheurer F. (2001). Balancing Device and Method.

[19] Aenis M., Knopf E., Nordmann R. (2002). Active magnetic bearings for the identification and fault diagnosis in turbomachinery. Mechatronics.

[20] Min J.B., Duffy K.P., Choi B.B., Provenza A., Kray N. Piezoelectric vibration damping study for rotating composite fan blades. Proceedings of the 14th AIAA Structures, Structural Dynamics, and Materials Conference.

[21] Simões R.C., Steffen V., Der Hagopian J., Mahfoud J. (2007). Modal active vibration control of a rotor using piezoelectric stack actuators. J. Vib. Control.

[22] Di Maio D., Ewins D. (2012). Experimental measurements of out-of-plane vibrations of a simple blisk design using blade tip timing and scanning LDV measurement methods. Mech. Syst. Signal Process..

[23] Procházka P., Vaněk F. Non-contact systems for monitoring blade vibrations of steam turbines. Proceedings of the International Conference on Noise and Vibration Engineering (ISMA).

[24] Zielinski M., Ziller G. (2000). Noncontact vibration measurements on compressor rotor blades. Meas. Sci. Technol..

[25] Georgiev V., Holík M., Kraus V., Krutina A., Kubín Z., Liška J., Poupa M. The blade flutter measurement based on the blade tip timing method. Proceedings of the 15th WSEAS International Conference on Systems.

[26] Di Maio D., Ewins D. (2010). Applications of continuous tracking SLDV measurement methods to axially symmetric rotating structures using different excitation methods. Mech. Syst. Signal Process..

[27] Staszewski W.J., bin Jenal R., Klepka A., Szwedo M., Uhl T. (2012). A Review of Laser Doppler Vibrometry for Structural Health Monitoring Applications. Key Eng. Mater..

[28] Polytec GmbH (2010). Theory Manual of Polytec Scanning Vibrometer.

[29] Boedecker S., Dräbenstedt A., Heller L., Kraft A., Leonhardt A., Pape C., Ristau S., Reithmeier E., Rembe C. (2006). Optical derotator for scanning vibrometer measurements on rotating objects. Proc. SPIE.

[30] Vibrometer P.S. (2008). Theory Manual.

[31] Hufenbach W., Kostka P., Filippatos A. (2013). Verfahren und Vorrichtung an Rotorsystemen.

[32] Philipp K., Filippatos A., Kuschmierz R., Langkamp A., Gude M., Fischer A., Czarske J. (2016). Multi-sensor system for in-situ shape monitoring and damage identification of high-speed composite rotors. Mech. Syst. Signal Process..

[33] Wollmann T., Filippatos A., Salles L., Lang T., Hoffmann N., Modler N. Development of a combined numerical and experimental approach for the 3D dynamic analysis of rotating components. Proceedings of the FVV 2018 Autumn Conference.

[34] Wollmann T., Modler N., Dannemann M., Langkamp A., Nitschke S., Filippatos A. (2017). Design and testing of composite compressor blades with focus on the vibration behaviour. Compos. Part A.

[35] Pickelmann L., Pickelmann L. (2003). Piezomechanics: An Introduction.

[36] Lobontiu N. (2002). Compliant Mechanisms: Design of Flexure Hinges.

[37] Cuntze R., Freund A. (2004). The predictive capability of failure mode concept-based strength criteria for multidirectional laminates. Compos. Sci. Technol..

